# Fatigue and cognitive dysfunction in previously hospitalized patients with COVID-19: A 1-year follow-up

**DOI:** 10.1371/journal.pone.0314131

**Published:** 2024-11-25

**Authors:** Ingrid Andreasson, Hanna C. Persson, Ann Björkdahl

**Affiliations:** 1 Department of Orthopedics, Clinical sciences, Sahlgrenska Academy, University of Gothenburg, Gothenburg, Sweden; 2 Department of Occupational Therapy and Physiotherapy, Sahlgrenska University Hospital, Gothenburg, Sweden; 3 Department of Clinical Neuroscience, Institute of Neuroscience and Physiology, Sahlgrenska Academy, University of Gothenburg, Gothenburg, Sweden; University of Rijeka Faculty of Medicine: Sveuciliste u Rijeci Medicinski fakultet, CROATIA

## Abstract

**Purpose:**

The aim was to longitudinally explore changes in fatigue- and cognition-related symptoms during the first year after hospital treatment for COVID-19.

**Method:**

Patients hospitalized for COVID-19 in Gothenburg, Sweden, were consecutively included from 01-07-2020 to 28-02-2021. Patients were assessed at the hospital (acute) and at 3 and 12 months after hospital discharge. Cognition was assessed with the Montreal Cognitive Assessment (MoCA), the Trail Making Test B (TMTB), and the Cognitive Failure Questionnaire (CFQ). Fatigue was assessed using the Multidimensional Fatigue Inventory-20 (MFI-20) and the Mental Fatigue Scale (MFS). Data was analyzed with demographics and changes over time calculated with univariable mixed-effects models.

**Result:**

In total, 122 participants were included. Analyzes of Z-scores for MoCA indicated improvement over the year, however the results were 1 SD below norm at all assessments. Alertness (TMTB scores) improved significantly from the acute assessment to the 12- month follow-up (*p* = <0.001, 95% CI 34.67–69.67). CFQ scores indicated cognitive impairment, and the sum scores for MFI reflected a relatively high degree of fatigue at follow-up.

**Conclusion:**

In the first year after hospitalization for COVID-19, most patients experienced fatigue and cognitive impairment. Alertness improved, but improvements in other domains were limited.

## Introduction

COVID-19 affects multiple organs with a variety of symptoms, and cases vary from being asymptomatic (about 20%) to involving severe illness and death [1]. A meta-analysis with over 1000 participants and 32 countries showed that at 12 months post-COVID infection 50% had at least one symptom still present, with the most common being memory impairment, fatigue, and depression [2]. Both patients who required hospital admission, including care in Intensive care unit (ICU), and those with a milder COVID-19 infection may have significant residual disabilities after a long period of time [3–5]. The condition with long-lasting problems was defined by the World Health Organization (WHO) as “post-COVID-19 condition” (PCC), which “occurs in individuals with a history of probable or confirmed SARS-CoV-2 infection, usually 3 months from the onset, with symptoms that last for at least 2 months and cannot be explained by an alternative diagnosis” [6]. A Swedish study using data from national registers and primary health care databases for all adult inhabitants of the two largest regions in Sweden (4.1 million inhabitants) found that of all registered COVID-19 cases, 2.0% (n = 10196) had been diagnosed with PCC [7].

It seems that different pathways are involved in the PCC sequelae, ranging from persistence of systemic inflammation, virus‐driven cellular alterations, dysregulated immune reaction, metabolic disturbances and coagulation and fibrosis pathways activation [8]. Structural and functional brain changes have been found after COVID-19, which provide insights to the understanding of neural underpinnings of cognitive dysfunction of PCC and the pathophysiology of this disorder [9–12].

Health status is generally poor for people with PCC, particularly in the domains of functional impairment (64%) and quality of life (72%) [13]. Fatigue and cognitive deficits are key features of PCC affecting daily activities for several patients over a long time [14]. A recent meta-analysis found fatigue to be the most frequent symptom of neurological post-COVID-19 syndrome (37%) followed by brain fog (32%), sleep disturbances (31%) and memory issues (28%) [15]. In a study of patients reporting major difficulties in everyday life following COVID-19, neuropsychological tests revealed cognitive impairment in 46%, whereas magnetic resonance imaging showed multiple areas of affected white matter in 71% [16]. Global impairment in cognition across the spectrum of COVID-19 disease severity has been shown, but no clear pattern of deficits in specific domains post-acute [17]. A recent study, with patients eight months post-COVID-19 infection, showed significantly lower performance on tests of attention, executive functions, and naming abilities, as well as subjective memory complaints of prospective and retrospective memory, with respect to a normative sample [18].

Fatigue is a multidimensional problem that is difficult to assess [19]. Studies of post-COVID fatigue focus on physical or mental fatigue or both using various scales, making results difficult to compare [20]. Cognitive impairment in people with post-COVID has been assessed with various neurological tests, but the most common screening is the Montreal Cognitive Assessment (MoCA) [21].

Fatigue and cognitive impairment limit everyday activities and cause disability that leads to the need for support and rehabilitation [13, 22]. People with PCC also can experience difficulties in understanding and managing their symptoms and in gaining insight from the healthcare system and accessing adequate rehabilitation efforts [23]. These difficulties emphasize the need for improved knowledge about the severity and progression of fatigue and cognitive impairment in people with post-COVID.

The aim of the present study was to longitudinally explore changes in fatigue- and cognition-related symptoms during the first year after hospital treatment for COVID-19.

## Materials and methods

### Patients

This longitudinal cohort study included patients enrolled in two studies, “Life in the time of Covid study in Gothenburg” (i.e., GOT-LOCO) and “Long-term Evaluation of the implication of COGnitive and other symptoms on everyday life” (i.e., LECOG-COVID-19). The two studies were coordinated regarding inclusion and follow-up. A total of 146 patients were consecutively included at Sahlgrenska University Hospital (four units), Gothenburg, Sweden, from 01-07-2020 to 28-02-2021 (first and second waves of the pandemic). Inclusion criteria were age ≥18 years and treatment in the hospital ≥5 days after (if needed) intensive care unit (ICU) treatment and a completed MoCA test during the hospital stay (designated here as the acute assessment). Exclusion criteria were cognitive or physical inability to perform cognitive screening, and not an independent living before the onset of COVID-19 infection.

Non-residents of Sweden were excluded. The study was approved by the Swedish Ethical Review authority (Drn: GOT-LOCO: 2020–03046, 2020–03922, 2021–00444, 2021–03556. LECOG: 2020–03222, 2021–03824) and complies with the Declaration of Helsinki. All patients signed written informed consent prior to inclusion. This manuscript was developed according to the STROBE guidelines [24].

### Procedure—data collection

A study coordinator reviewed medical records daily to recruit patients. Patients were subsequently informed and enrolled in the study by physiotherapists and occupational therapists at the hospital. The acute assessment was carried out at the hospital. The two in-person follow-ups were carried out at 3 and 12 months after hospital discharge. The follow-up was performed by an occupational therapist, mostly as an outpatient visit at the hospital or in the patient’s home if the patient could not travel. All assessments followed a standardized procedure and test protocol. Characteristics of the study population in the acute setting were retrived from the medical charts. Severity of COVID-19 was assessed according to the World Health Organizations Clinical Progression Scale [25, 26] moderate disease infection corresponding to hospital treatment with or without oxygen therapy (level 4–5), and severe disease was defined as needed more advanced respiratory or medical support (level 6–9) [25, 26]. For each patient the Charlson comorbidity index was calculated on data from the hospital medical records [27]. The Charlson comorbidity index is a weighted score, based on specific comorbid conditions, and was grouped as no comorbidity (0 points), mild comorbidity (1–2 points), and severe comorbidity (2 points).

#### Measurement of cognitive function and fatigue

Three measurements were used to assess different aspects of cognitive function. The MoCA is a brief screening test for cognitive impairment, with a maximum score of 30 points. Higher scores indicate less impairment, and a score <26 indicates cognitive impairment. The MoCA has high reliability and validity in patients with cognitive impairment [21]. To evaluate mental flexibility, speed of processing, and executive functions, the Trail Making Test B (TMTB) was used [28].

The Cognitive Failure Questionnaire (CFQ), consisting of 25 questions (each rated 0–4), was used for self-assessment of cognitive impairment. The total CFQ score range is 0–100, and higher scores indicate a worse degree of symptoms [29]. A standardized question also was used to explore whether participants experienced cognitive deficits that affected everyday life, with responses dichotomized as yes or no.

Three measurements were used to assess different aspects of fatigue. Self-perceived fatigue was assessed with the Swedish version of the Multidimensional Fatigue Inventory-20 (MFI-20). The MFI-20 includes five dimensions: general fatigue, physical fatigue, reduced activity, reduced motivation, and mental fatigue. Each dimension contains four items, each rated 0–5. A higher score indicates more fatigue, and the maximum total score is 100 points [30]. Self-perceived mental fatigue was assessed using the Mental Fatigue Scale (MFS), with 14 questions that are rated 0–3 and one question on daily variation that is rated 0–2. The score is based on the first 14 questions, and a score ≥10.5 points indicates mental fatigue in need of further examination [31]. The participants also rated fatigue on a visual analogue scale (VAS; 0–100 mm), where 100 mm indicated the worst possible fatigue. Furthermore, standardized questions were used to investigate the presence of sleepiness, lack of energy, and mental fatigue, and responses were dichotomized as yes or no.

The MoCA and TMTB, were used at all three timepoints and MFI-20, and MFS at 3 and 12 months. The CFQ was used only at the 12-month follow-up. Standardized questions regarding cognitive impact and different aspects of fatigue were asked at the 3- and 12-month follow-ups.

#### Statistical analysis

Data were analyzed using the Statistical Package for Social Sciences version 28.0 (IBM Corp., Armonk, NY, USA). Demographic and parametric data are presented as mean (standard deviation, SD), and non-parametric data as median [min, max]. To adjust for sex and age, Z-scores were calculated for MoCA [32], and t-scores were calculated for TMTB [33]. To investigate differences between groups, the Mann–Whitney U test was used. Two separate univariable mixed-effects models were used to evaluate changes in TMT-B and MoCA over time. The models were fitted using the lme function from the nlme package in R version 4.4.1 (R Foundation for Statistical Computing, Vienna, Austria), with random intercepts to account for individual variability across subjects. To address heteroscedasticity, separate variance structures were modelled for each time point.

## Results

**Fig 1:**
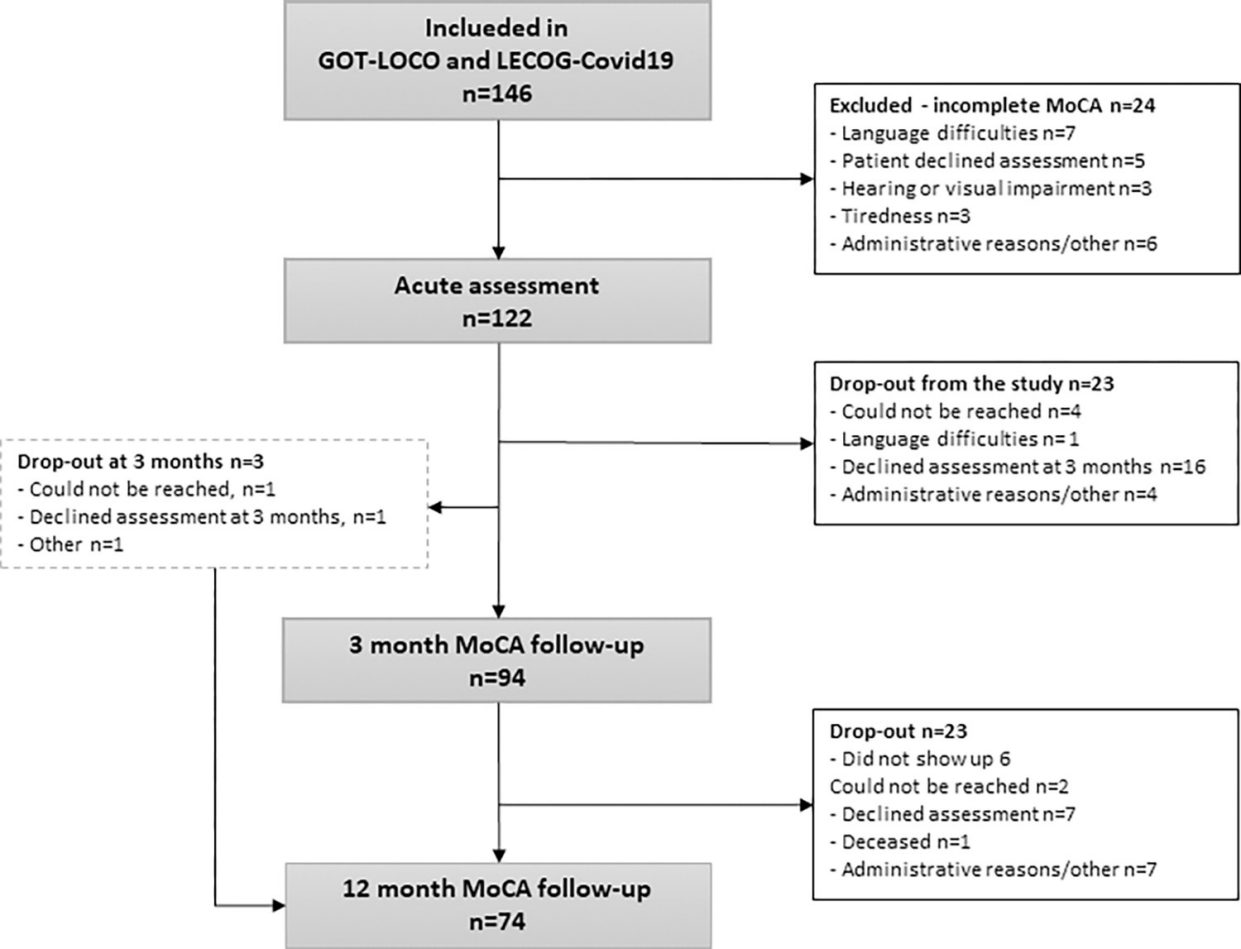
Flowchart of the inclusion process.

Of the 146 patients originally included in the studies, 122 met the inclusion criteria for the present study (Fig 1), with a mean age of 64 (standard deviation [SD] 13.2) years, and 75% were men. Most (70%) of the participants were born in Sweden, and almost half had an exam from secondary school (49%). All participants had been treated for COVID-19 in the hospital, for a mean of 40 (SD 42.1) days, including care in the ICU. More than half (55%) of the participants had been treated in the ICU (Table 1).

**Table 1:** Participant characteristics.

	Total sample
	*N* = 122
Age in years, mean (SD)	64 (13.2)
Men, n (%)	91 (75)
Length of hospital stay in days, mean (SD)	40 (42.1)
COVID-19 severity	
Moderate disease, n (%)	40 (33)
Severe disease, n (%)	82 (67)
Non-invasive mechanical ventilation, n (%) *(missing n = 5)*	73 (60)
Treated in ICU, n (%)	66 (55)
Length of stay in ICU, mean (SD)	23 (21.3)
Intubated, n (%)	
Need of tracheostomy, n (%)	34 (52%)
Comorbidities *(missing n = 6)*	
No comorbidity, n (%)	35 (30)
Mild comorbidity, n (%)	39 (33)
Severe comorbidity, n (%)	42 (37)
Education, n (%)	
Primary school	22 (18)
Secondary school	60 (49)
Post-secondary	40 (33)
Country of birth (missing *n* = 5), n (%)	
Sweden	82 (70)
Europe, except Sweden	23 (20)
Outside Europe	12 (10)

Abbreviations: ICU, intensive care unit; SD, standard deviation

A total of 94 participants attended the 3-month follow-up, and 74 attended at 12 months. Not all participants were assessed at both follow-ups, and some participated in only one assessment (Fig 1).

### Cognition

At the acute assessment, the median MoCA score was <26, indicating impaired function. The median score was 26 at both the 3- and 12-month follow-ups. When using the Z-scores for MoCA, i.e. considering age, sex, and education, the results were > 1 SD below the norm for the sample at all three assessments. Regarding TMT B at the acute assessment, 41% had a t-score <T40 (1 SD below the mean), and at the 12-month follow-up, 15% had a t-score <T40 (Table 2).

**Table 2:** Assessments of cognition at the acute, 3-month, and 12-month timepoints.

	Acute	3 months	12 months
**MoCA, *n***	*n* = 122	*n* = 94	*n* = 74
**and median [range] **	25 [8–30]	26 [10–30]	26 [14–30]
**MoCa z-score mean (SD) **	-1.67	-1.14	-1.05
	(SD 1.95)	(SD 1.75)	(SD 1.60)
**TMTB, *n* and**	*n* = 92	*n* = 90	*n* = 71
**mean (SD) [min–max] **	142 (SD 85)	107 (SD 68)	94 (SD 64)
	[30–372]	[22–440]	[24–317]
**TMTB, *n* and t-score**	*n* = 92	*n* = 90	*n* = 71
**mean (SD) [min–max] **	42 (SD 14.7) [-6–80]	48 (SD 14.8) [-28–80]	53 (SD 13.7) [20–80]
**CFQ, *n* and**			*n* = 62
**median [range] **			26 [4–80]
**Proportions **		*n* = 94	*n* = 79
**reporting cognitive**		64%	61%
**impairment, *n* and %**			

Abbreviations: confidence interval (CI), Cognitive Failure Questionnaire (CFQ), Montreal Cognitive Assessment (MoCA), Trail Making Test B (TMTB).

**Fig 2:**
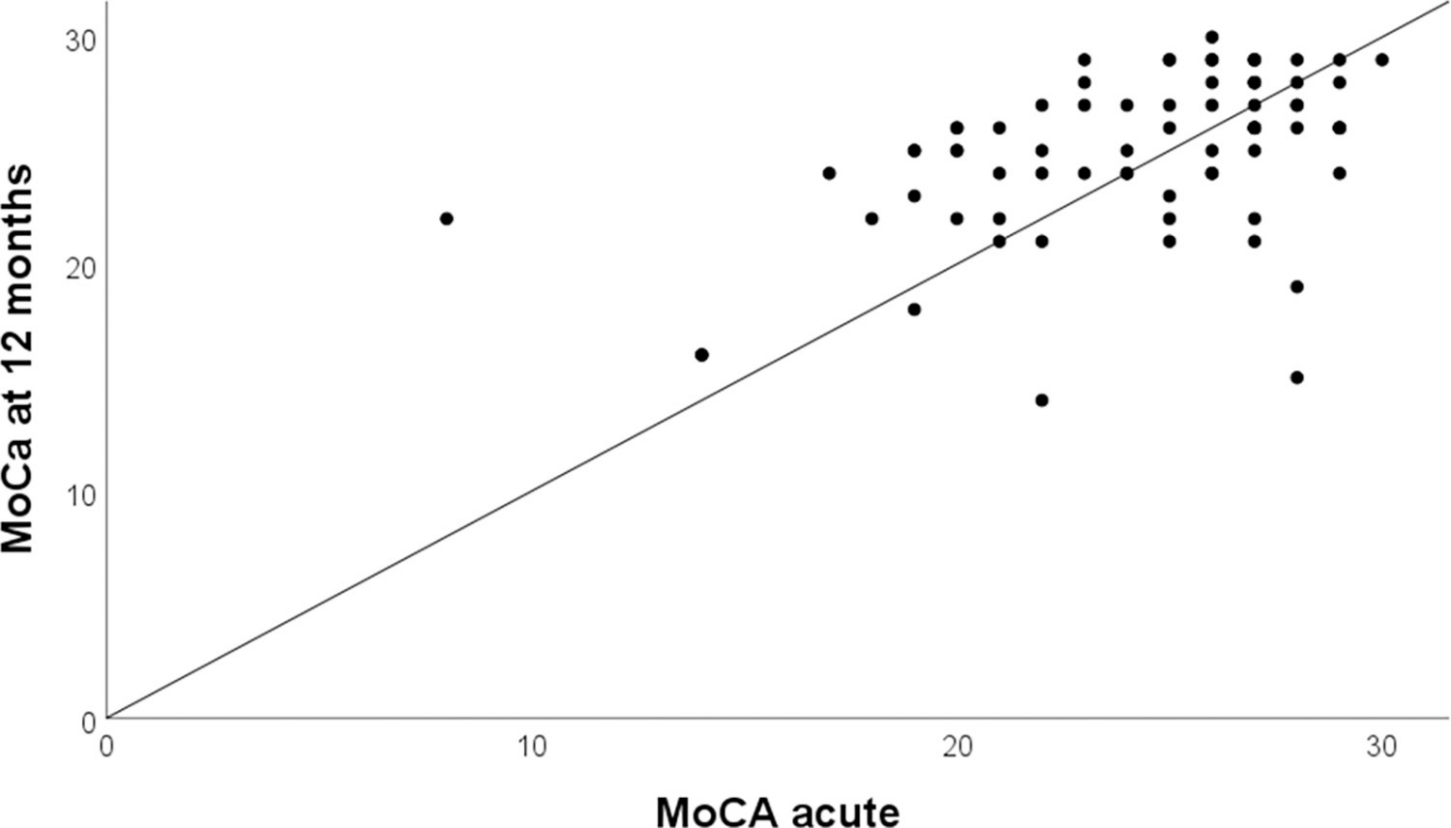
Montreal Cognitive Assessment (MoCA) scores at the acute assessment compared with scores at the 12-month follow-up (n = 74). Improvement is implied for values above the reference line (solid black line).

**Fig 3:**
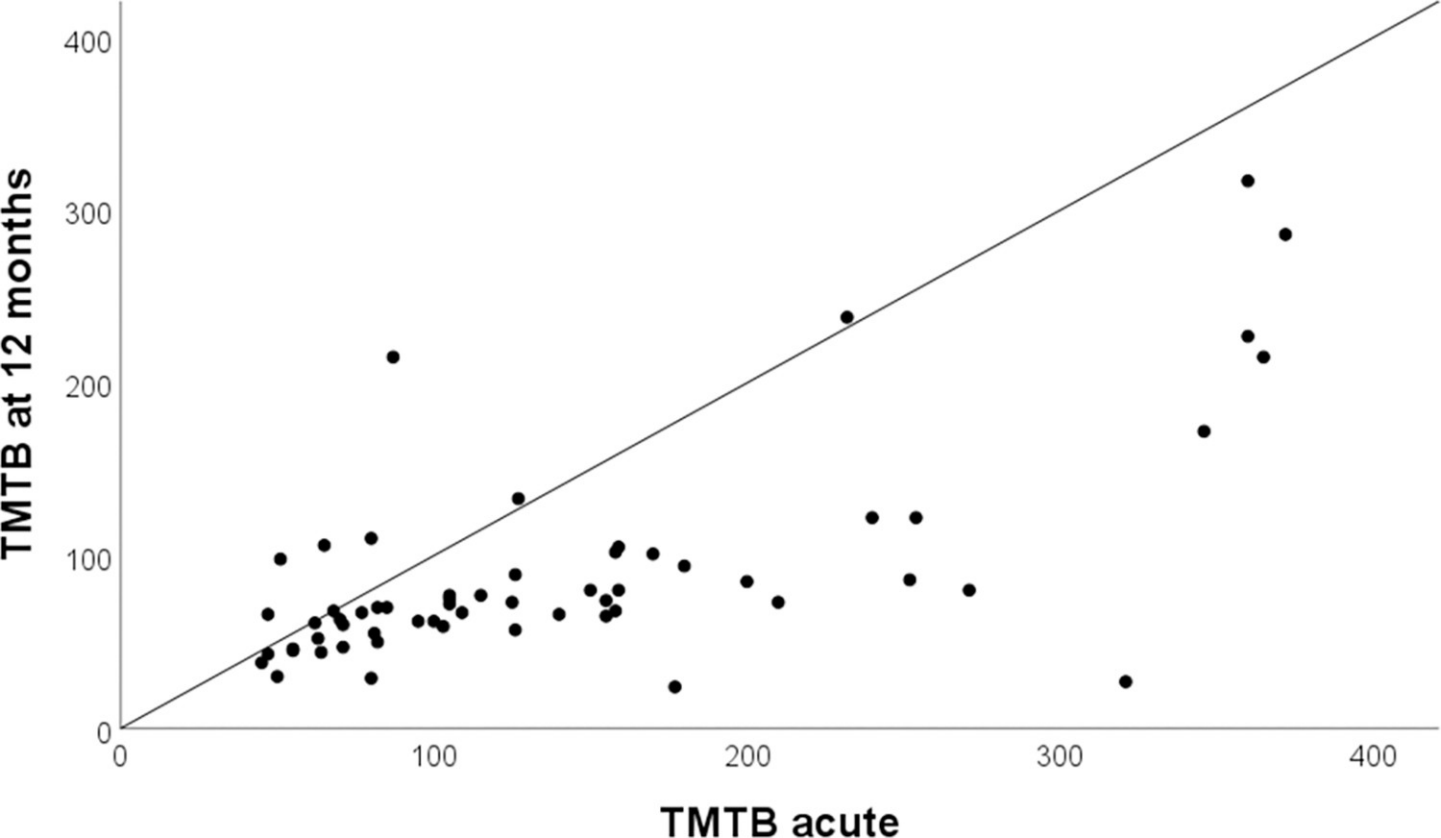
Trail Making Test B (TMTB) scores at the acute assessment compared with scores at 12 months (n = 71). Improvement is implied for values below the reference line.

The proportion of participants with an unchanged MoCA score between the acute and 3-month follow-up was 11%, whereas 35% had a worse score and 54% had an improved score. Between the acute assessment and the 12-month follow-up, 8% had no change, 37% had worse scores, and 55% had an improved score (Fig 2). For the TMTB scores, 86% had an improved score at the 12-month follow-up, 12% had a worse score, and for 2%, scores were unchanged (Fig 3).

Cognitive function (TMT B and MoCA) improved significantly over time, both at 3- and 12-months follow-up for both TMT B and MoCA (Table 3). Cognitive function (assessed with MoCA) did not differ significantly depending on treatment in the ICU or not.

**Table 3:** Change over time for the cognitive tests TMT B and MoCA.

	TMT-B			MoCA		
*Predictors *	*Estimates *	*CI *	*p *	*Estimates *	*CI *	*p *
(Intercept)	41.36	38.49–44.22	**<0.001 **	-1.67	-2.03–-1.31	**<0.001 **
t [3 months]	6.41	3.39–9.43	**<0.001 **	0.46	0.16–0.77	**0.003 **
t [12 months]	10.85	7.48–14.21	**<0.001 **	0.41	0.05–0.78	**0.026 **
**Random Effects **						
σ^2^	96.64			1.82		
τ_00_	106.78_id_			2.18_id_		
ICC	0.52			0.54		
N	110_id_			122_id_		
Observations	253			290		
Marginal R^2^/ Conditional R^2^	0.087 / 0.566			0.012 / 0.550		

Assessment with CFQ indicated experiences of cognitive impairment at 12 months (Table 2). Most problematic were questions related to attention and working memory (questions 2, 7, 9, 20, and 22). More than 20% of participants scored 3 or 4, indicating “quite often or often,” and <10% scored 0, meaning that they “never” experienced difficulties related to these questions.

Regarding the standardized question about the presence of cognitive impairment, 64% indicated experiencing cognitive impairment at 3 months and 61% did so at 12 months. The rates did not differ significantly between the two timepoints (Table 2).

### Fatigue

The level of fatigue (MFI) was reported as high (≥10 out of 20) in all but one of the dimensions at the 3- and 12-month follow-ups. The reported level of fatigue was approximately similar at both timepoints, with only “reduced activity” being less frequently reported at 12 months (Table 4).

**Table 4:** Fatigue at the 3- and 12-month follow-ups and changes between measurements.

	3 months	12 months	3 vs 12 months
MFI general	*n* = 96	*n* = 64	*p* = 0.231
Median [min–max]	14 [4–20]	13 [4–20]	z = -1.198
MFI physical	*n* = 96	*n* = 63	*p* = 0.132
Median [min–max]	15 [4–20]	13 [4–20]	z = -1.508
MFI mental	*n* = 96	*n* = 64	*p* = 0.666
Median [min–max]	10.5 [4–20]	10 [4–20]	z = -0.431
MFI reduced activity	*n* = 96	*n* = 64	*p* ≤ 0.001
Median [min–max]	13 [4–20]	12 [4–20]	z = -3.314
MFI reduced motivation	*n* = 96	*n* = 66	*p* = 0.861
Median [min–max]	8.5 [4–20]	8 [4–20]	z = -0.175
MFS		*n* = 72	
Median [min–max]		9 [0–32]	
Fatigue VAS mm	*n* = 92	*n* = 76	*p* = 0.270
Median [min–max]	49 [0–98]	41.5 [0–93]	z = -1.103
Mean (SD)	47.5 (SD 26.0)	41.8 (SD 25.7)	0.296 (95% CI -2.69–8.69)
Presence of sleepiness,	*n* = 93	*n* = 77	*p* = 0.414
n (%)	50 (54)	45 (58)	z = -0.82
Lack of energy,	*n* = 93	*n* = 77	*p* = 0.005
n (%)	79 (85)	53 (69)	z = -2.83
Presence of mental fatigue, n (%)	*n* = 91	*n* = 75	*p* = 0.394
	51 (56)	39 (52)	z = -0.85

Abbreviations: Multidimensional Fatigue Inventory-20 (MFI-20), Visual Analog Scale (VAS).

**Fig 4:**
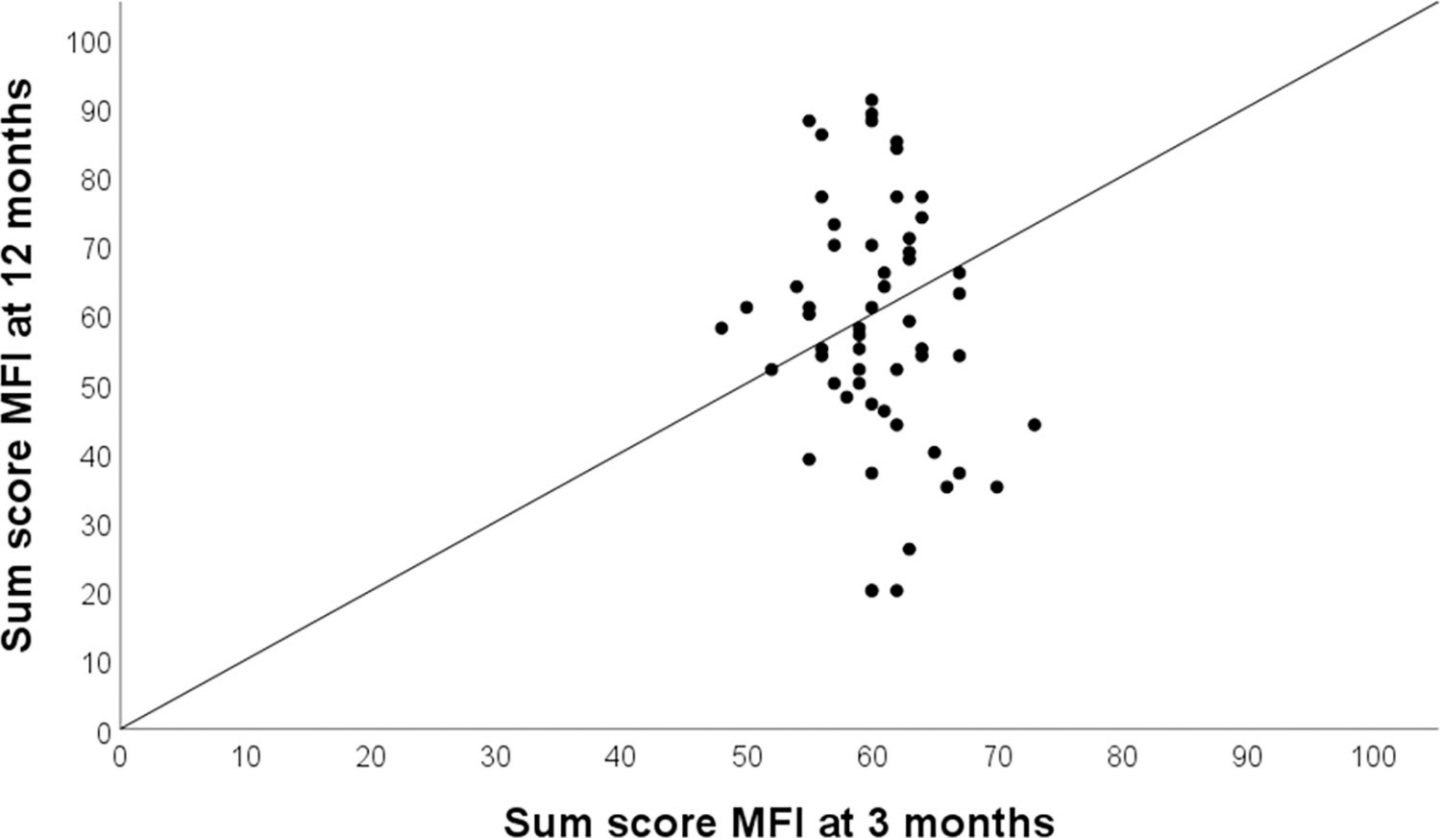
Change in Multidimensional Fatigue Inventory-20 (MFI-20) sum scores between the 3-month and 12-month follow-ups (n = 63). Improvement is implied for values below the reference line (solid black line).

Proportions of participants experiencing increased fatigue or reduced fatigue were similar at 12 months (Fig 4).

**Fig 5:**
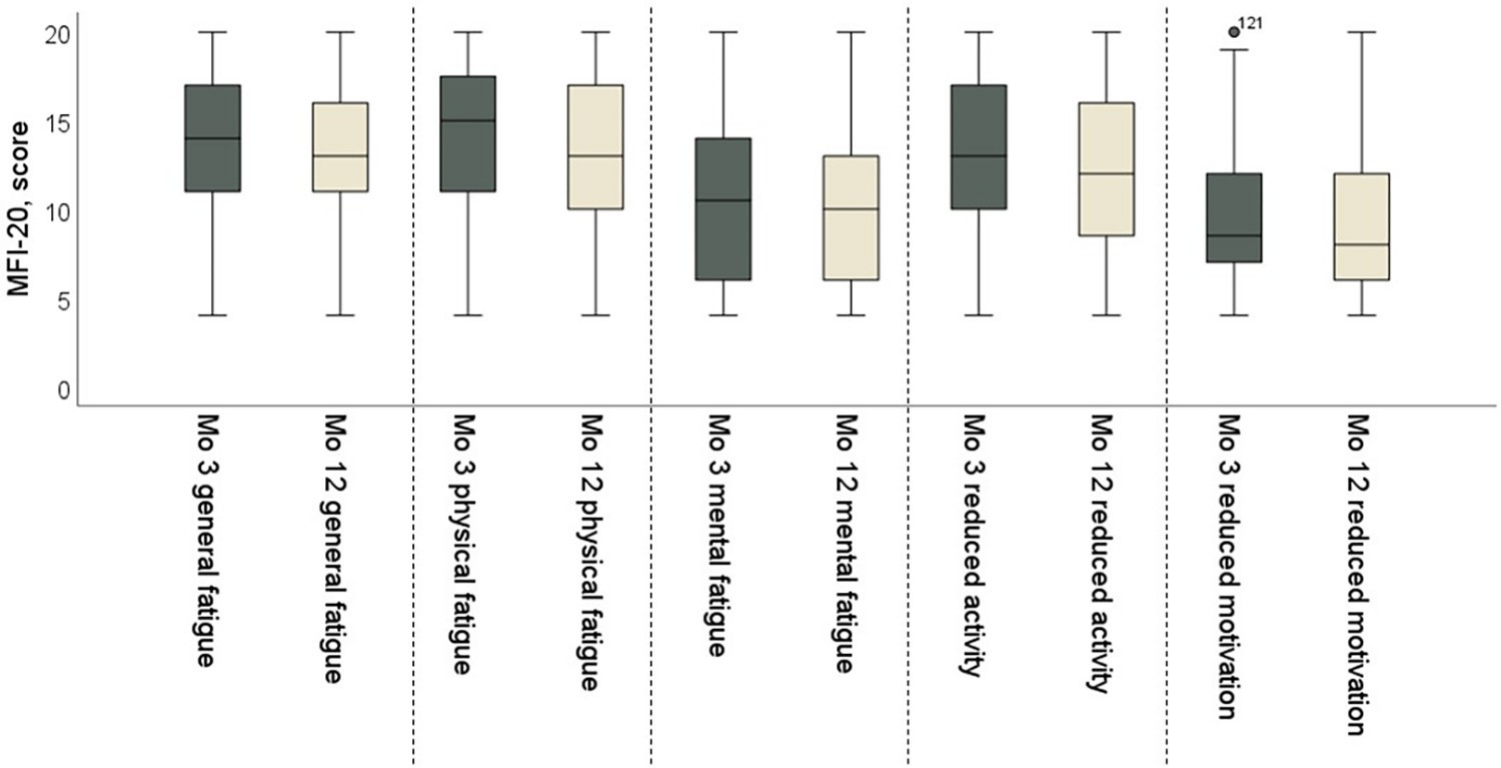
Changes in different dimensions of fatigue as assessed with the Multidimensional Fatigue Inventory-20 (MFI-20). Boxplots show the five dimensions of MFI-20 at the 3- and 12-month follow-ups.

The changes in fatigue for the total group of participants, assessed with MFI at 3 and 12 months, is illustrated in Fig 5. Fatigue (MFI dimension) did not differ with versus without ICU care at 12 months, except for “physical fatigue” scores, which were higher for patients who had not been treated in the ICU (*p* = 0.045, 95% CI -4.84 to -0.06).

**Fig 6:**
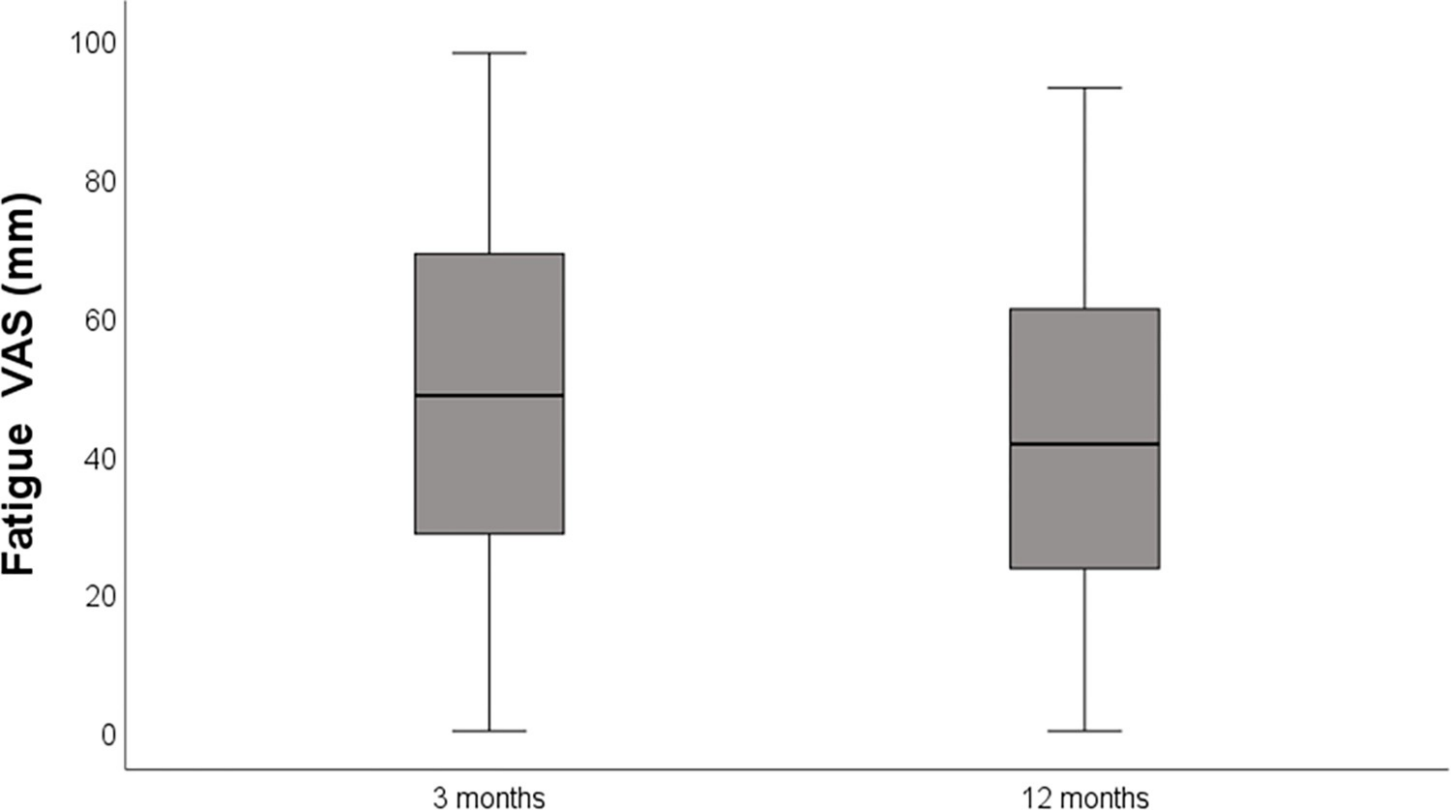
Boxplot showing the VAS (0–100 mm) fatigue ratings at the 3-month (*n* = 92) and 12-month follow-ups (*n* = 76). For each, the box represents interquartile ranges and contains 50% of the cases, the line represents the median, and the whiskers indicate the smallest and largest values.

When participants estimated fatigue using the VAS, the means were 48 (SD 26.0) at the 3-month follow-up and 42 (SD 25.7) at the 12-month follow-up (Table 3). The decrease was not significant (*p* = 0.297, 95% CI -2.69 to 8.69; Fig 6).

The median MFS score at 12 months was 9 [0–32] points. The proportion scoring ≥10.5, indicating mental fatigue, was 39%.

For the standardized question about presence of sleepiness, lack of energy, or mental fatigue, more than 50% stated that they had experienced all three at both 3 and 12 months (Table 4). At 3 months, 85% reported that they experienced a lack of energy, which had declined significantly to 69% at 12 months (*p* = 0.005, z = -2.83). For sleepiness and mental fatigue, the differences between 3 and 12 months were minimal (Table 4).

## Discussion

In this Swedish cohort study, among patients treated at the hospital for COVID-19, cognitive impairment was common within the first year. There was an improvement but results for global cognition (MoCA) was still >1 SD below norm at 12-months follow-up. Although alertness improved in the first year, which was indicated in improved results on TMT B, the improvement was insufficient to positively influence global cognitive function, which remained diminished. At one year after hospitalization for COVID-19, the prevalence of fatigue also was high.

MFI-scores indicated a higher degree of fatigue and below the norm for all other dimensions except for motivation [34]. This pattern might indicate that the motivation to be active had increased but that fatigue remained an obstacle. In line with our results, others have reported that fatigue may be persistent up to one year following COVID-19 [35–38]. Calabria et. al. [39] found that reports of clinically significant fatigue were present at eight months for most of the participants (82.3%) in their study, with physical fatigue rated highest, similar to the present study. We also found that mental fatigue (measured by MFS) was present in 4 of 10 participants at one year after COVID-19. Previous work from a post-COVID clinic in Primary care also has identified mental fatigue as one of the most severe consequences and a major persistent problem after COVID-19. Patients at this clinic needed special interventions because of residual symptoms, and the study categorized 75% of the participants as suffering from moderate to severe mental fatigue at about 9 months after infection [40]. Moreover, fatigue can reduce quality of life following COVID-19 [41].

Cognitive consequences after COVID-19 are common [42, 43], with persisting symptoms for one year or longer [44]. The current results indicated improvement in cognition during the year after COVID-19, on MoCA tests, calculated as z-scores, but based on self-reports of cognitive dysfunction the experience was that there was no improvement. Self-reported cognitive functions have been shown to correlate with the results of cognitive tests, and the experiences of cognitive difficulties persist confirmed by objective tests [43]. Although, even if there was an improvement in cognitive function the results were still below the general norm, which could correspond to the self-reported lack of improvement in cognitive function. A study in Italy assessed whether self-reports of cognitive impact could be verified neurophysiologically by altered electroencephalography patterns and showed a pattern characterized by a predominant slowing in 65% of cases [45]. Kopanska et. al. [10] compared Quantitative Electroencephalography (QEEG) findings before and after onset of PCC cognitive dysfunction and found objective changes in the QEEG profile in relation to the cognitive complaints. They concluded that a QEEG examination may be a supportive tool for post-COVID clinical workup and for monitoring the treatment effects.

Several studies of COVID-19 have used MoCA to explore the impact of the disease on global cognitive function, most using a cut-off score of <26 for impairment, regardless of age [5, 45–47]. However, age, sex, and level of education have been significantly associated with total MoCA score and are relevant to consider, rather than using a common cut-off [48]. For this reason, we performed z-score calculations, which could better be compared to normative data. The analyzes showed a small significant improvement between timepoints, but the results remained below the expected. There is an important difference between the reporting of results from raw scores as a mean at the cut -off (26 points) and using standard scores of Z, with a result for the sample of >-1SD meaning a performance below the norm, indicating that difficulties in daily life and work may be expected.

Our findings suggesting persistent problems are in line with a recent study of post-COVID patients treated in primary care, with results indicating neurocognitive disorders in more than 75% at 15 months after the infection [44]. However, flexibility and tempo, both aspects of cognitive function, were measured with the TMTB in the present study and showed significant improvement from the acute assessment to the 12-month follow-up, which is to be expected if alertness improves. Our results indicate that although the general cognitive impact persists, alertness increases, suggesting that to some extent, specific functions such as attention can be improved over time.

Fatigue and cognitive impairment are likely to affect each other, and they both seem to persist in the first year after COVID-19. Different symptoms in relation to long-term cognition have been explored, and fatigue has been identified in several domains associated with cognitive aspects [49]. Executive and attentional difficulties on cognitive tests have been found to be important predictors among different types of fatigue [39]. In PCC, neurocognitive symptoms and fatigue have also been associated with alterations in electroencephalographic (EEG) activity [9]. Cognitive impairment has considerable consequences for occupational performance, and fatigue also can heavily limit day-to-day activities [50] and be disabling [51, 52]. Even if the ability to work increases over time after COVID-19, fatigue and cognitive impairment may still greatly affect the ability to work [53]. Six months after infection, many patients need workplace accommodations or may not be able to resume their original employment [54–56]. According to one study of patients treated in the hospital for COVID-19, although most had returned to work by one year after infection, many still experienced various symptoms [57].

Given the great burden of post-COVID fatigue and cognitive impairment at both the individual and societal levels, the need is ongoing to investigate how long such problems persist or whether they will do so. Furthermore, effective interventions are needed to improve functioning and to discern which aspects of fatigue and cognitive impairment most affect the ability to perform activities of daily living. These aims are important to ensure the most appropriate rehabilitation measures that will allow people affected by COVID-19 to return to activity and work.

### Strengths and limitations

A strength of the present study is the longitudinal design, with the first assessment prior to discharge after in-hospital treatment for COVID-19 and follow-up at two timepoints during the first year after onset. As the study was conducted in an ongoing pandemic, however, rapid, and unforeseen ward changes became necessary for patients during their stay, so that it was difficult to adjust and carry out assessments as intended in a timely manner. For this reason, some data are missing, which is a limitation.

Another strength is that Z-scores were used for MoCA which not is the case in most COVID articles using MoCA. The most common is the use of raw sum score with adjustment of one point for participants with education less than 12 years. The use of z-scores takes into account sex, age, and level of education, offering a more solid assessment that does not have to rely on a cut-off which could be expected to be different in relation to sex, age and education. Furthermore, we used reliable and valid instruments complemented with self-ratings of fatigue and cognitive impairment to capture different aspects of these symptoms. For measuring cognitive impairment, however, more complete neuropsychological test batteries would have been preferable to a global screening instrument such as MoCA. As COVID was a new diagnosis, it was difficult to foresee that cognition would represent a significant residual impairment requiring deeper analysis. In hindsight, the choice of instruments would have been different to gain a deeper understanding of the impact of the disease on everyday life and work.

## Conclusion

In the first year after hospitalization with COVID-19, most patients in this study experienced fatigue and reduced cognitive function. Alertness improved over time, but other improvements were few. Because both fatigue and impaired cognitive function have a major impact on daily life, the results highlight the importance of further research with respect to treatment for these persistent problems, including rehabilitation.

## References

[1] Graham EL, Clark JR, Orban ZS, et al. Persistent neurologic symptoms and cognitive dysfunction in non-hospitalized Covid-19 "long haulers". Ann Clin Transl Neurol. 2021 May;8(5):1073–1085. doi: 10.1002/acn3.51350 33755344 PMC8108421

[2] Zeng N, Zhao YM, Yan W, et al. A systematic review and meta-analysis of long term physical and mental sequelae of COVID-19 pandemic: call for research priority and action. Mol Psychiatry. 2023 Jan;28(1):423–433. doi: 10.1038/s41380-022-01614-7 35668159 PMC9168643

[3] Steinmetz A, Gross S, Lehnert K, et al. Longitudinal Clinical Features of Post-COVID-19 Patients-Symptoms, Fatigue and Physical Function at 3- and 6-Month Follow-Up. J Clin Med. 2023 Jun 10;12(12). doi: 10.3390/jcm12123966 37373660 PMC10299126

[4] Chen C, Haupert SR, Zimmermann L, et al. Global Prevalence of Post-Coronavirus Disease 2019 (COVID-19) Condition or Long COVID: A Meta-Analysis and Systematic Review. J Infect Dis. 2022 Nov 1;226(9):1593–1607. doi: 10.1093/infdis/jiac136 35429399 PMC9047189

[5] Bungenberg J, Humkamp K, Hohenfeld C, et al. Long COVID-19: Objectifying most self-reported neurological symptoms. Ann Clin Transl Neurol. 2022 Feb;9(2):141–154. doi: 10.1002/acn3.51496 35060361 PMC8862437

[6] World Health Organization. Coronavirus disease (COVID-19): Post COVID-19 condition. 2021 [231129]. Available from: https://www.who.int/news-room/questions-and-answers/item/coronavirus-disease-(covid-19)-post-covid-19-condition.

[7] Bygdell M, Leach S, Lundberg L, et al. A comprehensive characterization of patients diagnosed with post-COVID-19 condition in Sweden 16 months after the introduction of the International Classification of Diseases Tenth Revision diagnosis code (U09.9): a population-based cohort study. Int J Infect Dis. 2023 Jan;126:104–113. doi: 10.1016/j.ijid.2022.11.021 36410693 PMC9678230

[8] Zuin M, Mazzitelli M, Cattelan A. Long-COVID: Is it time to revise the definition? J Med Virol. 2023 Aug;95(8):e29011. doi: 10.1002/jmv.29011 37526385

[9] Ortelli P, Quercia A, Cerasa A, et al. Lowered Delta Activity in Post-COVID-19 Patients with Fatigue and Cognitive Impairment. Biomedicines. 2023 Aug 8;11(8). doi: 10.3390/biomedicines11082228 37626724 PMC10452696

[10] Kopańska M, Ochojska D, Muchacka R, et al. Comparison of QEEG Findings before and after Onset of Post-COVID-19 Brain Fog Symptoms. Sensors (Basel). 2022 Sep 1;22(17). 10.3390/s22176606 PMC9460343 36081063

[11] Díez-Cirarda M, Yus M, Gómez-Ruiz N, et al. Multimodal neuroimaging in post-COVID syndrome and correlation with cognition. Brain. 2023 May 2;146(5):2142–2152. doi: 10.1093/brain/awac384 36288544 PMC9620345

[12] Wojcik GM, Shriki O, Kwasniewicz L, et al. Investigating brain cortical activity in patients with post-COVID-19 brain fog. Front Neurosci. 2023;17:1019778. doi: 10.3389/fnins.2023.1019778 36845422 PMC9947499

[13] van Kessel SAM, Olde Hartman TC, Lucassen P, et al. Post-acute and long-COVID-19 symptoms in patients with mild diseases: a systematic review. Fam Pract. 2022 Jan 19;39(1):159–167. doi: 10.1093/fampra/cmab076 34268556 PMC8414057

[14] Björkdahl A, Gustafsson M, Öhlén H, et al. Exploring the impact of cognitive dysfunction, fatigue, and shortness of breath on activities of daily life after COVID-19 infection, until 1-year follow-up. J Rehabil Med. 2024 Jun 25;56:jrm35403. doi: 10.2340/jrm.v56.35403 38915292 PMC11218676

[15] Premraj L, Kannapadi NV, Briggs J, et al. Mid and long-term neurological and neuropsychiatric manifestations of post-COVID-19 syndrome: A meta-analysis. J Neurol Sci. 2022 Mar 15;434:120162. doi: 10.1016/j.jns.2022.120162 35121209 PMC8798975

[16] Hellgren L, Birberg Thornberg U, Samuelsson K, et al. Brain MRI and neuropsychological findings at long-term follow-up after COVID-19 hospitalisation: an observational cohort study. BMJ Open. 2021 Oct 27;11(10):e055164. doi: 10.1136/bmjopen-2021-055164 34706965 PMC8551746

[17] Fanshawe JB, Sargent BF, Badenoch JB, et al. Cognitive domains affected post-COVID-19; a systematic review and meta-analysis. Eur J Neurol. 2024 Feb 20:e16181. 10.1111/ene.16181 PMC11618111 38375608

[18] Arbula S, Pisanu E, Bellavita G, et al. Insights into attention and memory difficulties in post-COVID syndrome using standardized neuropsychological tests and experimental cognitive tasks. Sci Rep. 2024 Feb 22;14(1):4405. doi: 10.1038/s41598-024-54613-9 38388708 PMC10883994

[19] Rudroff T, Fietsam AC, Deters JR, et al. Post-COVID-19 Fatigue: Potential Contributing Factors. Brain Sci. 2020 Dec 19;10(12). doi: 10.3390/brainsci10121012 33352638 PMC7766297

[20] Rao S, Benzouak T, Gunpat S, et al. Fatigue Symptoms Associated With COVID-19 in Convalescent or Recovered COVID-19 Patients; a Systematic Review and Meta-Analysis. Ann Behav Med. 2022 Mar 1;56(3):219–234. doi: 10.1093/abm/kaab081 34665858 PMC8574547

[21] Nasreddine ZS, Phillips NA, Bedirian V, et al. The Montreal Cognitive Assessment, MoCA: a brief screening tool for mild cognitive impairment. J Am Geriatr Soc. 2005 Apr;53(4):695–9. doi: 10.1111/j.1532-5415.2005.53221.x 15817019

[22] Hellgren L, Levi R, Divanoglou A, et al. Seven Domains of Persisting Problems after Hospital-treated Covid-19 Indicate a Need For a Multiprofessional Rehabilitation Approach. J Rehabil Med. 2022 Jul 25;54:jrm00301. doi: 10.2340/jrm.v54.2434 35678268 PMC9422323

[23] Kingstone T, Taylor AK, O’Donnell CA, et al. Finding the ’right’ GP: a qualitative study of the experiences of people with long-COVID. BJGP Open. 2020 Dec;4(5). 10.3399/bjgpopen20X101143 PMC7880173 33051223

[24] von Elm E, Altman DG, Egger M, et al. The Strengthening the Reporting of Observational Studies in Epidemiology (STROBE) statement: guidelines for reporting observational studies. Lancet. 2007 Oct 20;370(9596):1453–7. doi: 10.1016/S0140-6736(07)61602-X 18064739

[25] Correction to Lancet Infect Dis 2020; 20: e192-97. Lancet Infect Dis. 2020 Oct;20(10):e250. 10.1016/S1473-3099(20)30637-X PMC7423329 32800100

[26] A minimal common outcome measure set for COVID-19 clinical research. Lancet Infect Dis. 2020 Aug;20(8):e192–e197. doi: 10.1016/S1473-3099(20)30483-7 32539990 PMC7292605

[27] Charlson ME, Pompei P, Ales KL, et al. A new method of classifying prognostic comorbidity in longitudinal studies: development and validation. J Chronic Dis. 1987;40(5):373–83. doi: 10.1016/0021-9681(87)90171-8 3558716

[28] Espenes J, Hessen E, Eliassen IV, et al. Demographically adjusted trail making test norms in a Scandinavian sample from 41 to 84 years. Clin Neuropsychol. 2020 Dec;34(sup1):110–126. doi: 10.1080/13854046.2020.1829068 33034252

[29] Broadbent DE, Cooper PF, FitzGerald P, et al. The Cognitive Failures Questionnaire (CFQ) and its correlates. Br J Clin Psychol. 1982 Feb;21(1):1–16. doi: 10.1111/j.2044-8260.1982.tb01421.x 7126941

[30] Smets EM, Garssen B, Bonke B, et al. The Multidimensional Fatigue Inventory (MFI) psychometric qualities of an instrument to assess fatigue. J Psychosom Res. 1995 Apr;39(3):315–25. doi: 10.1016/0022-3999(94)00125-o 7636775

[31] Johansson B, Starmark A, Berglund P, et al. A self-assessment questionnaire for mental fatigue and related symptoms after neurological disorders and injuries. Brain Inj. 2010 Jan;24(1):2–12. doi: 10.3109/02699050903452961 20001478

[32] Borland E, Nägga K, Nilsson PM, et al. The Montreal Cognitive Assessment: Normative Data from a Large Swedish Population-Based Cohort. Journal of Alzheimers Disease. 2017;59(3):893–901. doi: 10.3233/JAD-170203 28697562 PMC5545909

[33] Tombaugh TN. Trail Making Test A and B: normative data stratified by age and education. Arch Clin Neuropsychol. 2004 Mar;19(2):203–14. doi: 10.1016/S0887-6177(03)00039-8 15010086

[34] Engberg I, Segerstedt J, Waller G, et al. Fatigue in the general population- associations to age, sex, socioeconomic status, physical activity, sitting time and self-rated health: the northern Sweden MONICA study 2014. BMC Public Health. 2017 Aug 14;17(1):654. doi: 10.1186/s12889-017-4623-y 28806984 PMC5557471

[35] Ceban F, Ling S, Lui LMW, et al. Fatigue and cognitive impairment in Post-COVID-19 Syndrome: A systematic review and meta-analysis. Brain Behav Immun. 2022 Mar;101:93–135. doi: 10.1016/j.bbi.2021.12.020 34973396 PMC8715665

[36] Townsend L, Dyer AH, Jones K, et al. Persistent fatigue following SARS-CoV-2 infection is common and independent of severity of initial infection. PLoS One. 2020;15(11):e0240784. doi: 10.1371/journal.pone.0240784 33166287 PMC7652254

[37] Stavem K, Ghanima W, Olsen MK, et al. Prevalence and Determinants of Fatigue after COVID-19 in Non-Hospitalized Subjects: A Population-Based Study. Int J Environ Res Public Health. 2021 Feb 19;18(4). doi: 10.3390/ijerph18042030 33669714 PMC7921928

[38] Mirfazeli FS, Sarabi-Jamab A, Pereira-Sanchez V, et al. Chronic fatigue syndrome and cognitive deficit are associated with acute-phase neuropsychiatric manifestations of COVID-19: A 9-month follow-up study. Neurol Sci. 2022 Apr;43(4):2231–2239. doi: 10.1007/s10072-021-05786-y 35059902 PMC8776380

[39] Calabria M, García-Sánchez C, Grunden N, et al. Post-COVID-19 fatigue: the contribution of cognitive and neuropsychiatric symptoms. J Neurol. 2022 Aug;269(8):3990–3999. doi: 10.1007/s00415-022-11141-8 35488918 PMC9055007

[40] Nielsen TB, Leth S, Pedersen M, et al. Mental Fatigue, Activities of Daily Living, Sick Leave and Functional Status among Patients with Long COVID: A Cross-Sectional Study. Int J Environ Res Public Health. 2022 Nov 9;19(22). doi: 10.3390/ijerph192214739 36429458 PMC9690484

[41] Garrigues E, Janvier P, Kherabi Y, et al. Post-discharge persistent symptoms and health-related quality of life after hospitalization for COVID-19. J Infect. 2020 Dec;81(6):e4–e6. doi: 10.1016/j.jinf.2020.08.029 32853602 PMC7445491

[42] Guo P, Benito Ballesteros A, Yeung SP, et al. COVCOG 2: Cognitive and Memory Deficits in Long COVID: A Second Publication From the COVID and Cognition Study. Front Aging Neurosci. 2022;14:804937. doi: 10.3389/fnagi.2022.804937 35370620 PMC8967943

[43] Crivelli L, Palmer K, Calandri I, et al. Changes in cognitive functioning after COVID-19: A systematic review and meta-analysis. Alzheimers Dement. 2022 May;18(5):1047–1066. doi: 10.1002/alz.12644 35297561 PMC9073922

[44] Van Wambeke E, Bezler C, Kasprowicz AM, et al. Two-Years Follow-Up of Symptoms and Return to Work in Complex Post-COVID-19 Patients. J Clin Med. 2023 Jan 17;12(3). doi: 10.3390/jcm12030741 36769389 PMC9917586

[45] Furlanis G, Buoite Stella A, Biaduzzini F, et al. Cognitive deficit in post-acute COVID-19: an opportunity for EEG evaluation? Neurol Sci. 2023 May;44(5):1491–1498. doi: 10.1007/s10072-023-06615-0 36749529 PMC9902820

[46] Solaro C, Gamberini G, Masuccio FG. Cognitive impairment in young COVID-19 patients: the tip of the iceberg? Neurol Sci. 2021 Dec;42(12):4865–4866. 10.1007/s10072-021-05534-2 PMC8369866 34403027

[47] Del Brutto OH, Rumbea DA, Recalde BY, et al. Cognitive sequelae of long COVID may not be permanent: A prospective study. Eur J Neurol. 2022 Apr;29(4):1218–1221. doi: 10.1111/ene.15215 34918425

[48] Borland E, Nägga K, Nilsson PM, et al. The Montreal Cognitive Assessment: Normative Data from a Large Swedish Population-Based Cohort. J Alzheimers Dis. 2017;59(3):893–901. doi: 10.3233/JAD-170203 28697562 PMC5545909

[49] Ariza M, Cano N, Segura B, et al. COVID-19 severity is related to poor executive function in people with post-COVID conditions. J Neurol. 2023 May;270(5):2392–2408. doi: 10.1007/s00415-023-11587-4 36939932 PMC10026205

[50] Bilgin A, Kesik G, Ozdemir L. ’The body seems to have no life’: The experiences and perceptions of fatigue among patients after COVID-19. J Clin Nurs. 2021 Nov 29. doi: 10.1111/jocn.16153 34845774

[51] Jason LA, Taylor RR, Kennedy CL. Chronic fatigue syndrome, fibromyalgia, and multiple chemical sensitivities in a community-based sample of persons with chronic fatigue syndrome-like symptoms. Psychosom Med. 2000 Sep-Oct;62(5):655–63. doi: 10.1097/00006842-200009000-00009 11020095

[52] Yorkston KM, Johnson K, Boesflug E, et al. Communicating about the experience of pain and fatigue in disability. Qual Life Res. 2010 Mar;19(2):243–51. doi: 10.1007/s11136-009-9572-1 20033786 PMC2844855

[53] Gualano MR, Rossi MF, Borrelli I, et al. Returning to work and the impact of post COVID-19 condition: A systematic review. Work. 2022;73(2):405–413. doi: 10.3233/WOR-220103 35938280

[54] Carenzo L, Dalla Corte F, Haines RW, et al. Return to Work After Coronavirus Disease 2019 Acute Respiratory Distress Syndrome and Intensive Care Admission: Prospective, Case Series at 6 Months From Hospital Discharge. Crit Care Med. 2021 Nov 1;49(11):e1157–e1162. doi: 10.1097/CCM.0000000000005096 34048368 PMC8507591

[55] van Veenendaal N, van der Meulen IC, Onrust M, et al. Six-Month Outcomes in COVID-19 ICU Patients and Their Family Members: A Prospective Cohort Study. Healthcare (Basel). 2021 Jul 8;9(7). doi: 10.3390/healthcare9070865 34356243 PMC8305246

[56] Lindahl A, Aro M, Reijula J, et al. Women report more symptoms and impaired quality of life: a survey of Finnish COVID-19 survivors. Infect Dis (Lond). 2022 Jan;54(1):53–62. doi: 10.1080/23744235.2021.1965210 34410220

[57] Huang L, Yao Q, Gu X, et al. 1-year outcomes in hospital survivors with COVID-19: a longitudinal cohort study. Lancet. 2021 Aug 28;398(10302):747–758. doi: 10.1016/S0140-6736(21)01755-4 34454673 PMC8389999

